# Identification of a new genetic variant (G231N, E232T, N235D) of peptidylarginine deiminase from *P. gingivalis* in advanced periodontitis

**DOI:** 10.3389/fimmu.2024.1355357

**Published:** 2024-03-21

**Authors:** Grzegorz P. Bereta, Karolina Strzelec, Katarzyna Łazarz-Bartyzel, Agata Dziedzic-Kowalska, Zuzanna Nowakowska, Anna Krutyhołowa, Ewa Bielecka, Tomasz Kantyka, Aleksander M. Grabiec, Tomasz Kaczmarzyk, Maria Chomyszyn-Gajewska, Jan Potempa, Katarzyna Gawron

**Affiliations:** ^1^ Malopolska Centre of Biotechnology, Jagiellonian University, Krakow, Poland; ^2^ Department of Molecular Biology and Genetics, Faculty of Medical Sciences in Katowice, Medical University of Silesia, Katowice, Poland; ^3^ Department of Periodontology, Preventive Dentistry and Oral Pathology, Faculty of Medicine, Medical College, Jagiellonian University, Krakow, Poland; ^4^ Department of Microbiology, Faculty of Biochemistry, Biophysics and Biotechnology, Jagiellonian University, Krakow, Poland; ^5^ Department of Oral Surgery, Medical College, Jagiellonian University, Krakow, Poland; ^6^ Department of Oral Immunology and Infectious Diseases, School of Dentistry, University of Louisville, Louisville, KY, United States

**Keywords:** chronic periodontitis, P. gingivalis, virulence, peptidylarginine deiminase, polymorphic variant(s), genetic variability, disease prognosis

## Abstract

Chronic periodontitis (CP), an inflammatory disease of periodontal tissues driven by a dysbiotic subgingival bacterial biofilm, is also associated with several systemic diseases, including rheumatoid arthritis (RA). *Porphyromonas gingivalis*, one of the bacterial species implicated in CP as a keystone pathogen produces peptidyl arginine deiminase (PPAD) that citrullinates C-terminal arginine residues in proteins and peptides. Autoimmunity to citrullinated epitopes is crucial in RA, hence PPAD activity is considered a possible mechanistic link between CP and RA. Here we determined the PPAD enzymatic activity produced by clinical isolates of *P. gingivalis*, sequenced the *ppad* gene, and correlated the results with clinical determinants of CP in patients from whom the bacteria were isolated. The analysis revealed variations in PPAD activity and genetic diversity of the *ppad* gene in clinical *P. gingivalis* isolates. Interestingly, the severity of CP was correlated with a higher level of PPAD activity that was associated with the presence of a triple mutation (G231N, E232T, N235D) in PPAD in comparison to W83 and ATCC 33277 type strains. The relation between mutations and enhanced activity was verified by directed mutagenesis which showed that all three amino acid residue substitutions must be introduced into PPAD expressed by the type strains to obtain the super-active enzyme. Cumulatively, these results may lead to the development of novel prognostic tools to assess the progress of CP in the context of associated RA by analyzing the *ppad* genotype in CP patients infected with *P. gingivalis*.

## Introduction

Chronic periodontitis (CP) is a highly prevalent infection-driven inflammatory disease, estimated to affect over 40% of the population of 30 years old and over 60% of population older than 65 years in the USA (1, 2). Prolonged inflammation of the gingiva results in pathologic processes that lead to the destruction of periodontal tissue and eventually tooth loss (3). *Porphyromonas gingivalis* (*P. gingivalis*) is considered a “keystone” bacterial species involved in the establishment and progression of periodontitis (4, 5). In brief, this bacterium has been found to induce shifts in the microbiota of subgingival biofilm from commensal to dysbiotic and to disable host antibacterial defenses (6, 7). Factors responsible for the pathogenicity of *P. gingivalis* include cysteine proteinases, called gingipains, specific for arginine-Xaa (RgpA and RgpB) and lysine-Xaa (Kgp) peptide bonds (8, 9); fimbriae that facilitate attachment to host cells (10, 11); and the enzyme peptidylarginine deiminase (PPAD), which modifies C-terminal arginine residues in peptide chains (12).

Deimination, also known as citrullination of an arginine residue with the release of ammonium ion, results in the loss of a positive charge from the amino acid side chain and can therefore lead to alterations in the structure of modified peptides/proteins. Although PPAD and mammalian peptidylarginine deiminases (PADs) catalyze identical biochemical reactions and have similar structures and modes of action, they are genetically unrelated. PPAD differs from PADs in its preference for C-terminal residues, ability to modify free arginine, and independence from calcium ions (13–15). PPAD has been found to affect the virulence of *P. gingivalis* by inactivating or diminishing the biological activities of several host proteins, such as epidermal growth factor (EGF) and complement protein C5a, leading to disturbances in the processes of wound healing and immune responses, respectively (16, 17). Moreover, active PPAD was shown to be crucial for the effective adhesion of *P. gingivalis* to primary human gingival fibroblasts (PHGFs) and for bacterial invasion of these cells. PPAD was also shown to stimulate the production of prostaglandin E_2_ (PGE_2_), a mediator of inflammation involved in periodontal bone resorption (18). Furthermore, PPAD alters the production of outer membrane vesicles, which are loaded with *P. gingivalis* virulence factors (19). Citrullination has also been regarded as a possible mechanism of breaching immunological tolerance, leading to rheumatoid arthritis (RA), as PPAD can modify several human proteins, such as fibrinogen, α-enolase, vimentin and histones (20–22). This aspect of *P. gingivalis* virulence is further highlighted by the ability of arginine-specific gingipains (RgpA and B) to generate substantial amounts of arginine-terminated peptides, with these products of protein cleavage being the preferred substrates for PPAD. Antibodies targeting RgpA and PPAD have been proposed as biomarkers of RA, and peptides with C-terminal citrulline can stimulate T-cells, leading to autoimmunity (23, 24). The hypothesis that PPAD action may lead to the development of RA is also supported by the active enzyme presence in gingival crevicular fluid (GCF) samples from patients with CP (25).

*P. gingivalis* clinical strains obtained from patients diagnosed with CP display a high degree of genetic diversity due to multiple mechanisms of genetic material exchange (26). Genetic diversity leads to the variable pathogenic potential of individual *P. gingivalis* strains, as demonstrated by *in vitro* adhesion/invasion studies (27). Variations at the genetic level are also observed in virulence factors expressed by this periodontopathogen, with the *fimA* gene, which encodes the major fimbriae protein, being the most studied example of diversity to date. Six types of this protein, which vary in human cell invasion activity, have been described (28, 29). FimA types II and IV are the types most frequently observed in CP donors (30). Moreover, distinct types of the gingipains RgpA, RgpB and Kgp, which differ in catalytic domain structure, have been described, although this variability has not been associated with differences in the virulence of *P. gingivalis (* 31–33). Additionally, possible variations of the *ppad* gene were investigated in CP patients with and without co-existing RA, but no relevant differences were found (34). Nevertheless, abundant PPAD expression in all analyzed clinical strains of *P. gingivalis* reported in another study highlights the importance of this protein as one of the crucial primary virulence factors of this periodontopathogen (35).

The present study therefore investigated the genetic variability in *ppad* gene sequences from *P. gingivalis* clinical strains and assessed the potential associations between the clinical characteristics of CP and the presence and virulence of novel genetic PPAD variant(s).

## Materials and methods

### Study groups

The study was carried out in accordance with the Declaration of Helsinki and was approved by the Bioethics Committee of Jagiellonian University Medical College in Krakow, Poland (KBET/310/B/2012 and 1072.6120.156.2019). Prior to the study, written informed consent was obtained from all donors. The study group consisted of 30 donors diagnosed with CP (18 women, 12 men; age range 37-73 years), whereas the control group consisted of 15 donors in good general health without periodontitis as indicated by the lack of pathological pockets (PPD > 3 mm and CAL >2 mm) (11 women, 4 men; age range 21-62 years). Subjects were excluded if they had been diagnosed with a medical condition that required pre-medication/pre-treatment prior to dental visits; had five or more decayed untreated teeth at screening; had been diagnosed with other diseases of the hard or soft oral tissues; had taken antibiotics or antimicrobial drugs within 30 days prior to the first visit; had a history of systemic disease, including RA, aspiration pneumonia, diabetes mellitus, atherosclerosis or other uncharacterized systemic disease; were pregnant or lactating; were tobacco smokers; or were immune-compromised individuals.

### Clinical and periodontal examination

Clinical examinations and sample collection were performed at the Department of Periodontology, Preventive Dentistry and Oral Pathology, Jagiellonian University Medical College in Krakow, Poland, in 2014 and 2015. Medical, dental, and medication history was recorded by the clinician. The diagnosis of CP was confirmed by radiography and periodontal examination of each patient.

### Sample collection

Study participants were asked to refrain from eating or drinking, excluding water, beginning at 11:00 pm on the night before sample collection and to brush their teeth in the evening before the visit, but not on the morning of collection. This procedure was applied to prevent disruption of subgingival microflora and facilitated GCF collection and subsequent isolation of *P. gingivalis.* Pooled samples of GCF were collected from five gingival pockets within areas of visible signs of inflammation (defined by redness, swelling and gums bleeding) in each subject with CP and from five random gingival crevices in each control subject. The probing pocket depth (PPD) and clinical attachment level (CAL) of gingival pockets affected by the inflammatory process were measured. GCF was sampled from deep pockets and used for *P. gingivalis* isolation. The same clinical parameters were determined for five random gingival crevices per control subject to verify their periodontal health.

### *P. gingivalis* clinical strains culturing from GCF

GCF samples collected from CP and healthy subjects were diluted with sterile phosphate-buffered saline (PBS) (1:2) and grown on blood agar plates (BHI, brain heart infusion) supplemented with 5% defibrinated sheep blood (Proanimali), 5 g·L^-1^ yeast extract (BioShop), 500 μg·mL^-1^ L-cysteine (BioShop), 10 µg·mL^-1^ hemin (Sigma-Aldrich), and 0.5 µg·mL^-1^ menadione (Sigma-Aldrich) in an anaerobic chamber (85% N2, 10% CO2, and 5% H2) for 14 days. After incubation, black pigmented colonies were streaked on fresh blood agar plates, with streaking repeated every 7 days until homogeneous cultures of *P. gingivalis* were obtained. These cultures were suspended in BHI with 20% glycerol and stored at -80°C.

### *P. gingivalis* genomic DNA isolation

Clinical isolates of *P. gingivalis* cultured on blood agar plates for 7 days were inoculated into enriched BHI broth and cultured overnight. To isolate bacterial genomic DNA, 2 × 10^9^ bacterial cells were collected (an optical density of 1.0 at 600 nm corresponds to 1 × 10^9^ bacterial cells per 1 mL) and genomic DNA was purified using GeneJET Genomic DNA Purification Kits (Thermo Scientific).

### Genotyping of *P. gingivalis* clinical strains

DNA purified from clinical isolates of *P. gingivalis* was eluted with 50 µL of ultra-sterile water and used in PCR reactions to verify species genotype. Each sample was subjected to two PCR reactions, one targeting the *P. gingivalis 16S rRNA* gene and the other targeting the full *ppad* gene (**Table 1**). Each reaction mixture contained DreamTaq Green PCR Master Mix (Thermo Scientific) and 2 µL of template DNA. The amplification protocol consisted of an initial denaturation at 95°C for 3 minutes; 40 cycles of denaturation at 95°C for 30 seconds, annealing at 60°C for 30 seconds, and extension at 72°C for 60 seconds (*16S rRNA*) or 2 minutes (*ppad*); followed by a final extension at 72°C for 5 minutes. PCR reaction products were separated on 1.5% agarose gels, which were stained with ethidium bromide. The presence of specific products of both PCR reactions was required to confirm *P. gingivalis* species.

**Table 1:** Primer sequences used in the study (synthetized by Genomed SA or Sigma-Aldrich).

Primer name	Sequences
*P. gingivalis* 16S rRNA	FOR: AGGCAGCTTGCCATACTGCG REV: ACTGTTAGYAACTACCGATGT
Full *PPAD*	FOR: ATGAAAAAGCTTTTACAGGCTAAAGCCTTG REV: TTATTTGAGAATTTTCATTGTCTCACGGATTCC
pUC_19	FOR: GAGCTCGGTACCCGGGGATC REV: GAATTCACTGGCCGTCGTTTTACAACG
Frag_A	FOR: ACGGCCAGTGAATTCTTTACGGGCGGTTATCGGG REV: GAGATAATTCGTTGTATTAAGAATATCAGTGGAGAAAATAATAC
Frag_B	FOR: ACAACGAATTATCTCCTTAACG REV: CCGGGTACCGAGCTCCTCCGTATAGAGCAGGATC
PPAD_T2	FOR: AACAATACTTATATCGACCATGTGGACTGTTGGGGCAAGTATTTGGC REV: CACATGGTCGATATAAGTATTGTTCGGATCTTGTACCACATCATGATGTGTGATGC
PPAD_G231N	FOR: AACAATGAATATATCAACCATGTGGACTGTTGGGGCAAGTATTTGGC REV: CACATGGTTGATATATTCATTGTTCGGATCTTGTACCACATCATGATGTGTGATGC
PPAD_E232T	FOR: AACGGCACTTATATCAACCATGTGGACTGTTGGGGCAAGTATTTGGC REV: CACATGGTTGATATAAGTGCCGTTCGGATCTTGTACCACATCATGATGTGTGATGC
PPAD_N235D	FOR: AACGGCGAATATATCGACCATGTGGACTGTTGGGGCAAGTATTTGGC REV: CACATGGTCGATATATTCGCCGTTCGGATCTTGTACCACATCATGATGTGTGATGC
PPAD_6HisI	FOR: ATGCATCACCATCATCACCACAACGAAACCAATACATGTACTGTGACC REV: GTTGTGGTGATGATGGTGATGCATACACGTAAACTTGAAAGGATCAGGTTC

### Cloning and sequencing of *PPAD*

Complete *P. gingivalis ppad* gene sequence was amplified with Phusion polymerase in HF buffer (Thermo Scientific) using *ppad* gene primers (**Table 1**), and 200 ng of genomic DNA as a template. The amplification protocol consisted of an initial denaturation at 98°C for 30 seconds; 40 cycles of denaturation at 98°C for 10 seconds, annealing at 61°C for 15 seconds, and extension at 72°C for 1 minute; followed by a final extension at 72°C for 5 minutes. PCR products were separated in 1% agarose gels and extracted with GeneJET Gel Extraction Kits (Thermo Scientific). Adenine overhangs were added by incubating the purified products in PCR mix with Taq polymerase, and modified products were purified using GeneJET PCR Purification Kits (Thermo Scientific). The resulting *ppad* sequences were cloned into pTZ57R/T plasmid using InsTAclone PCR Cloning Kits (Thermo Scientific), and the obtained plasmid was used to transform *E. coli* DH5α. Single *E. coli* colonies were inoculated into 5 mL of LB broth and incubated overnight with shaking at 37°C. Three plasmids per isolate were purified from cultures with GeneJET Plasmid Miniprep Kit (Thermo Scientific) and sequenced by Genomed S.A. (Warsaw, Poland) using the standard primers M13fwd and M13rev. The *ppad* gene sequences from clinical *P. gingivalis* strains were compared with the ATCC 33277 reference strain and other sequences deposited in GenBank database using NCBI and BLAST (36). Accession numbers of sequences obtained in this study and used for analysis are listed in **Supplementary Table 1**.

### Peptidylarginine deiminase activity assay

To test the activity of PPAD, *P. gingivalis* clinical strains were cultured. Briefly, bacterial cultures were inoculated into supplemented, liquid BHI medium and incubated in an anaerobic chamber at 37°C for 18 – 20 hours. Following incubation, the OD_600_ of cultures was measured and new cultures in fresh medium were started from OD_600_ = 0.1 and incubated for 16 – 18 hours. The final OD_600_ of the cultures was measured and aliquots were withdrawn to assess PPAD activity. The remainder of each culture was washed twice in PBS and adjusted to OD_600_ = 1 in PBS, and PPAD activity was evaluated. To assay activity, 10 µL of each sample was mixed with 40 µL of activity buffer (100 mM Tris – HCl, pH 7.5, 5 mM dithiothreitol, 10 mM N-acetyl-L-arginine) and incubated for 1 hour at 37°C. The reaction was stopped by the addition of 10 µL of 5 M perchloric acid. The produced citrulline was measured using a colorimetric method and compared with a standard curve for L-citrulline. Fresh citrulline detection reagent was prepared immediately before use by mixing 1 part of solution A (0.5% 2,3-butanedione monoxime, 0.01% thiosemicarbazide) with 2 parts of solution B (0.25 mg·mL^-1^ iron[III] chloride, 24.5% sulphuric acid, 17% orthophosphoric acid), with 150 µL aliquots added to each analyzed and standard curve sample. The samples were incubated at 110°C for 20 minutes, and the absorbance of each sample at 535 nm was measured; the amount of citrullinated product was calculated using the standard curve.

### Evaluation of *ppad* mRNA expression level

Liquid cultures of *P.gingivalis* strains were prepared as described above, except that 1 mL aliquots were withdrawn when the OD_600_ of the culture reached 1. The samples were centrifuged at 5000 × g for 5 minutes at 4°C, and the bacteria were resuspended in 500 µL of Trizol solution (Thermo Scientific) and incubated for 20 minutes at 60°C. Total RNA was isolated according to the manufacturer’s instructions. Aliquots containing 2 µg of each purified RNA were incubated with 2 U of Turbo DNAse (Thermo Scientific) for 1 hour at 37°C, followed by the addition of 500 µL of Trizol solution and a repeat RNA isolation. Finally, 50 ng of RNA were reverse transcribed using High-Capacity cDNA Reverse Transcription Kit (Thermo Scientific). Real-time PCR was performed using 2 µL of cDNA in 10 µL reaction mixture containing iTaq Universal SYBR Green Supermix (BioRad) and 0.5 µM of each primer. The primer sequences for 16S rRNA were 5’-AGGCAGCTTGCCATACTGCG-3’ (forward) and 5’-ACTGTTAGYAACTACCGATGT-3’ (reverse), and the primer sequences for *ppad* were 5’-TGTACGATACGAACAAAGTAGGTC-3’ (forward) and 5’-AATACTTGCCCCAACAGTCCAC-3’ (reverse). The amplification protocol consisted of an initial denaturation at 95°C for 3 minutes; followed by 40 cycles of denaturation at 95°C for 30 seconds, and annealing and extension at 60°C for 20 seconds. Data were analyzed with BioRad CFX Manager using ATCC 33277 as a reference sample.

### Introduction of PPAD T2 or point mutations into *P. gingivalis* ATCC 33277

**Figure 1:**
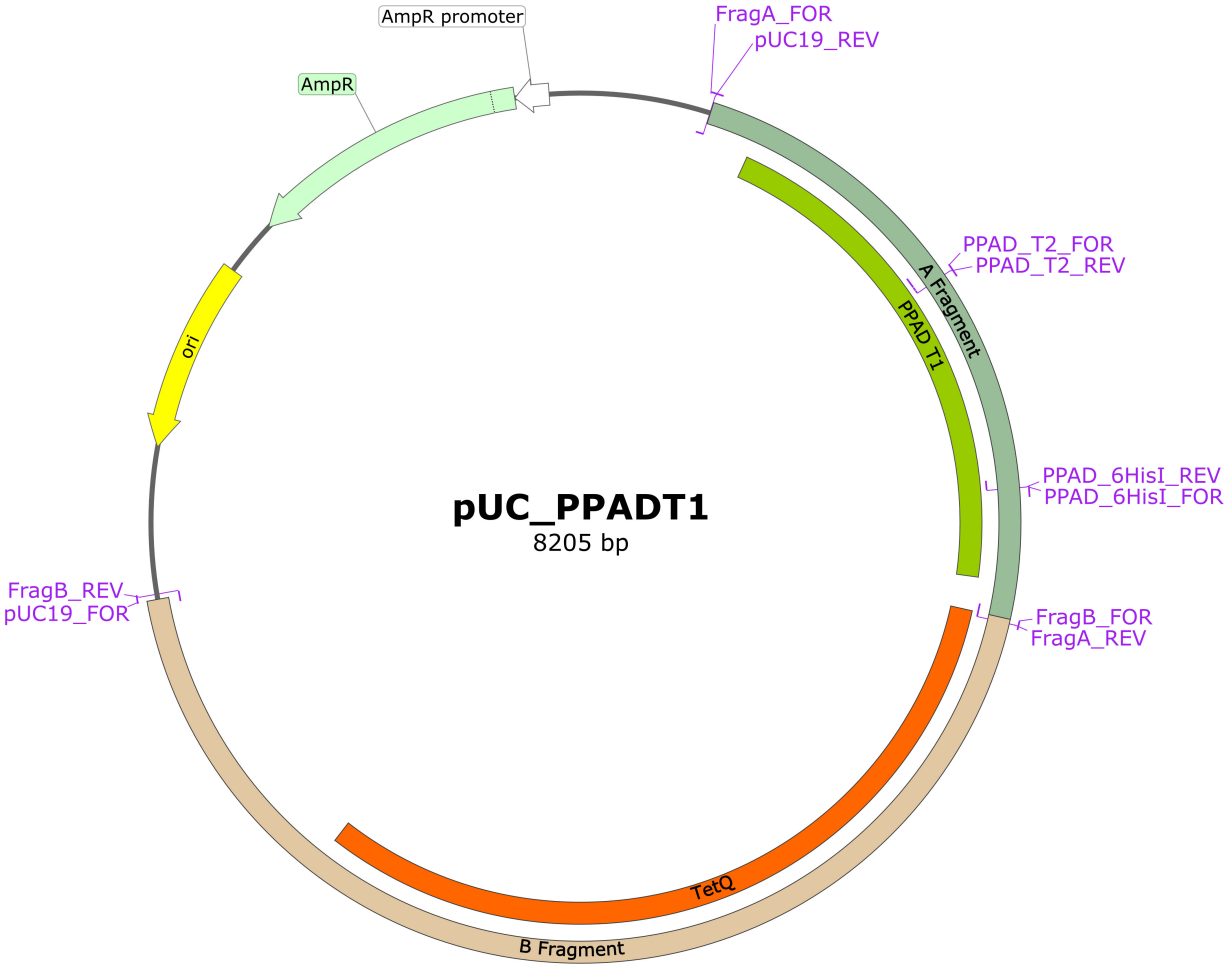
Schematic map of plasmid pUC_PPADT1 used for obtaining other plasmids for mutagenesis. Regions marked as A and B fragment contain at their ends sequences flanking *ppad* gene in the genome of *P. gingivalis* and allow recombination, introducing mutated *ppad* and TetQ resistance cassette from the plasmid. Mutations were introduced into this plasmid by linearization in PCR with indicated primer pairs and reassembly using overlapping mutated regions. Primers for mutations G231N, E232T and N235D bind in the same region as primers PPAD_T2 and are not marked on the map. Map was prepared with SnapGene^®^ software (from Dotmatics; available at Available at: snapgene.com).

Mutations corresponding to the PPAD T2 variant or point mutations were introduced into the reference *P. gingivalis* ATCC 33277 strain using standard procedures, as described elsewhere (37, 38). The pUC19 vector was linearized with the pUC19 primers. The A fragment (FragA primers) spanning the *ppad* gene with flanking sequences was amplified from the genomic DNA of the ATCC 33277 strain, and the B fragment (FragB primers) spanning the tetracycline resistance cassette and the sequence downstream of the *ppad* gene was amplified from the genomic DNA of a C351A strain (18). All products were separated in agarose gels, excised, and purified. These fragments were subsequently assembled into the final plasmid using Gibson Assembly Master Mix (New England Biolabs), according to the manufacturer’s instructions. The plasmid was used to transform *E. coli* DH5α and the resulting pUC_PPADT1 plasmid was purified as described. Schematic map of the plasmid is presented in **Figure 1**, ends of A and B fragments allow recombination with genome of *P. gingivalis* to replace the native *ppad* gene with mutated variants together with TetQ tetracycline resistance cassette. Point mutations corresponding to the PPAD T2 variant were introduced into the plasmid by amplification of the pUC_PPADT1 plasmid with the FragA_FOR + PPAD_T2_REV and PPAD_T2_FOR + pUC19_REV primers, followed by digestion with DpnI enzyme. The resulting products were purified, assembled, and used to transform DH5α, as described. Single point mutations (G231N, E232T, N235D, and hexahistidine tag insertion for protein purification) were introduced by amplifying the plasmid pUC_PPADT1 with corresponding primer pairs, which introduced mutations into overlapping ends of linearized plasmid allowing recircularization when introduced into *E. coli*. Reaction products were digested with the enzyme DpnI, used to transform *E. coli* DH5α and purified. The sequences of all primers used in the mutagenesis procedure are presented in **Table 1** and on the plasmid map (**Figure 1**). Finally, the sequences of the constructed plasmids were verified by sequencing of the *ppad* gene (Genomed S.A., Warsaw, Poland).

Electrocompetent *P. gingivalis* ATCC 33277 cells were prepared as described by Bélanger et al (37). Briefly, liquid overnight culture of ATCC 33277 was used to inoculate fresh cultures with starting OD_600_ = 0.1 in TSB medium (tryptic soy broth 30 g·L^-1^, yeast extract 5 g·L^-1^ supplemented with 500 µg·mL^-1^ L-cysteine, 5 µg·mL^-1^ hemin and 1 µg·mL^-1^ menadione) and cultured in an anaerobic chamber until OD_600_ = 0.55 – 0.65. The cultures were centrifuged, and the bacteria were washed twice in 1 mM magnesium chloride with 10% glycerol and stored at -80°C in the same buffer. These bacteria were subsequently electroporated with a 1 µg sample of each mutagenic plasmid at 2.5 kV for 4 miliseconds, followed by the addition of 1 mL of fresh TSB medium to each sample and incubation overnight in an anaerobic chamber. These bacteria were plated onto BHI blood agar plates supplemented with tetracycline (1 µg·mL^-1^) and incubated anaerobically for 14 days. Single colonies were picked and streaked onto fresh plates, and the occurrence of mutations was assessed by *ppad* sequencing.

### PPAD purification from mutated *P. gingivalis* ATCC 33277 strains

Proteins were purified from strains of *P. gingivalis* producing PPAD with various mutations and a hexahistidine tag inserted in front of the C-terminal domain (39). Briefly, bacteria were cultured and inoculated as described above, except that the concentration of hemin was 2.5 µg·mL^-1^. A 40 mL aliquot of culture was inoculated into 900 mL of fresh medium, followed by incubation for 20-24 hours in an anaerobic chamber. The cultures were centrifuged at 6500 x g for 25 minutes at 4°C, and the supernatants were collected and cooled on ice. Proteins were precipitated by addition of chilled acetone to a 60% concentration and centrifugation at 10000 g for 20 minutes at 4°C. PPAD was purified by affinity chromatography on Ni-NTA resin, followed by size exclusion chromatography on a S75 column and ion exchange on a MonoQ column.

### Enzyme kinetic assays

Kinetic assays were performed as described in the previous study (38). Enzymes at concentrations of 1.5 nM were incubated in reaction buffer (100 mM Tris, 5 mM DTT, pH 7.5) with increasing concentrations of peptide substrate Eno: IHAREIFDSR with N-terminal 5-FAM fluorophore. Samples were taken at each time point and added to 0.5% trifluoroacetic acid (TFA) to stop the reaction. Peptides were resolved on an Aeris 3.6 μm Peptide XB-C18 100Å (150 × 4.6 mm) column (Phenomenex) connected to a Shimadzu HPLC system with fluorescence detection. Samples were loaded in the presence of 2% phase B (80% acetonitrile, 0.08% TFA in water) in phase A (0.1% TFA in water) and resolved on a linear gradient of 35 – 50% phase B over 12 column volumes.

## Results

### Identification of *P. gingivalis* clinical strains by *16S rRNA* and *ppad* expression analysis

**Figure 2:**
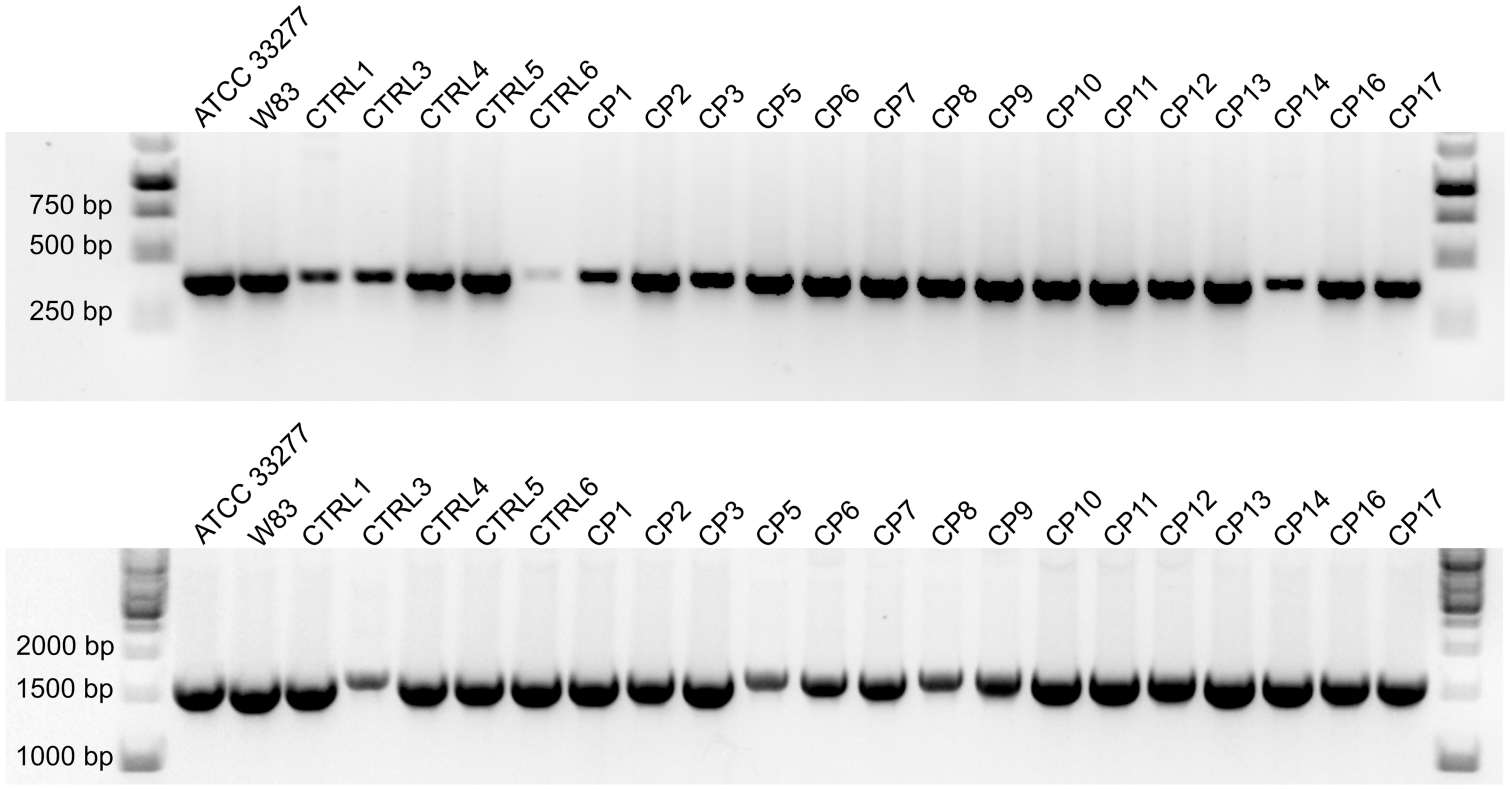
Agarose gel electrophoresis of PCR products from reactions specific for the *16S rRNA* (top) and *ppad* (bottom) genes of *P. gingivalis*. Results from representative clinical strains isolated from control and CP groups are shown; laboratory strains ACTT 33277 and W83 were used as positive controls.

*P. gingivalis* strains were successfully cultured from GCF samples collected from 23 of the 30 subjects with CP and 8 of the 15 controls, with one clinical strain obtained from each donor. *P. gingivalis* clinical strains were identified by PCR amplification of the *16S rRNA* and *ppad* genes, with the latter being unique to this species. PCR products were detected for all strains, with representative results depicted in **Figure 2**.

### Diagnosis and clinical classification of chronic periodontitis

CP diagnosis was confirmed by dental history, radiography and clinical examination of the periodontium. Of the 23 *P. gingivalis-*positive subjects with CP enrolled in this study, 2, 12, 4 and 5 were classified as having mild, moderate, moderate/advanced and advanced periodontitis, respectively. Subjects in good general health and without periodontitis were included in control group (**Table 2**).

**Table 2:** Clinical characteristics of study participants and classification of periodontal disease.

CP group					
*n=23	Age		Gender		Clinical classification of periodontal disease
	Range	Mean	Females	Males	
	37 – 73	55.9	14	9	
n=2	44 – 56	50	2	0	mild
n=12	37 – 73	54.5	6	6	moderate
n=4	49 – 69	61.5	3	1	moderate/advanced
n=5	47 – 71	57.2	3	2	advanced
Control group					
	Age		Gender		
	Range	Mean	Females	Males	
*n = 8	21 – 29	25.1	7	1	Healthy periodontium

*Clinical presentation defined as CP and healthy subjects culture positive for *P. gingivalis* strains.

### Analysis of *ppad* gene sequence reveals significant diversity among *P. gingivalis* strains from individual subjects with CP

Bioinformatic analysis revealed that the *ppad* gene sequences in clinical isolates collected from subjects with CP had significantly greater heterogeneity compared to the ATCC 33277 P*. gingivalis* type strain. **Table 3** summarizes the synonymous variants, missense mutations and polymorphic variants of the *ppad* gene in the CP and control groups. Of the 51 nucleotide substitutions identified in the CP group, 35 showed as synonymous variants and 16 resulted in sequence modifications of the encoded proteins. Ten of these 16 substitutions were missense mutations, and six were classified as polymorphic variants. In addition, 7 synonymous and 4 polymorphic variants were in close proximity to the active center, being located near His236 and Asn297. Of the 27 nucleotide substitutions identified in the control group, the majority constituted synonymous variants (22), 5 missense mutations, but polymorphic variants were not found. **Table 4** shows an overview of nucleotide substitutions in the *ppad* gene in each individual CP and control subjects.

**Table 3:** Summarized analysis of synonymous variants, missense mutations and polymorphic variants in the CP and Control groups.

Analyzed group	Total changes of PPAD	Total synonymous variants [frequency]	Synonymous variants in close proximity to the active center of the PPAD [frequency]	Total missense mutations [frequency]	Missense mutations in close proximity to the active center of the PPAD [frequency]	Total polymorphic variants [frequency]	Polymorphic variant in close proximity to the active center of the PPAD [frequency]
CP group [n=23]	51	35 [68. 63%]	7 [13. 72%]	10 [19. 61%]	0 [0%]	6 [11. 76%]	4 [7. 84%]
Ctrl group [n=8]	27	22 [81. 48%]	4 [14. 81%]	5 [18. 52%]	4 [14. 81%]	0 [0%]	0 [0%]

**Table 4:** Overview of nucleotide substitutions in the *ppad* gene per individual CP (n=23) and Control subject (n=8).

Study group			
CP donors [n=23]	Total synonymous variants	Total missense mutations	Total polymorphic variants
CP1	0	0	0
CP2	5	2	0
CP3	11	0	0
CP4	11	0	0
CP5	9	0	0
CP6	11	0	4
CP7	12	2	0
CP8	11	0	2
CP9	14	2	5
CP10	9	2	2
CP11	10	2	4
CP12	13	3	4
CP13	10	1	1
CP14	2	0	0
CP15	14	1	2
CP16	10	1	1
CP17	9	0	0
CP18	11	0	2
CP19	10	1	2
CP20	11	0	3
CP21	10	2	4
CP22	8	0	4
CP23	7	0	0
Control group			
Healthy donors [n=8]	Total synonymous variants	Total missense mutations	Total polymorphic variant
CTRL1	0	0	0
CTRL2	11	0	0
CTRL3	0	0	0
CTRL4	12	0	0
CTRL5	9	0	0
CTRL6	0	0	0
CTRL7	11	3	0
CTRL8	11	2	0

### Identification of a new polymorphic variant (G231N, E232T, N235D) of the *ppad* gene in subjects with moderate/advanced and advanced periodontitis

**Figure 3:**
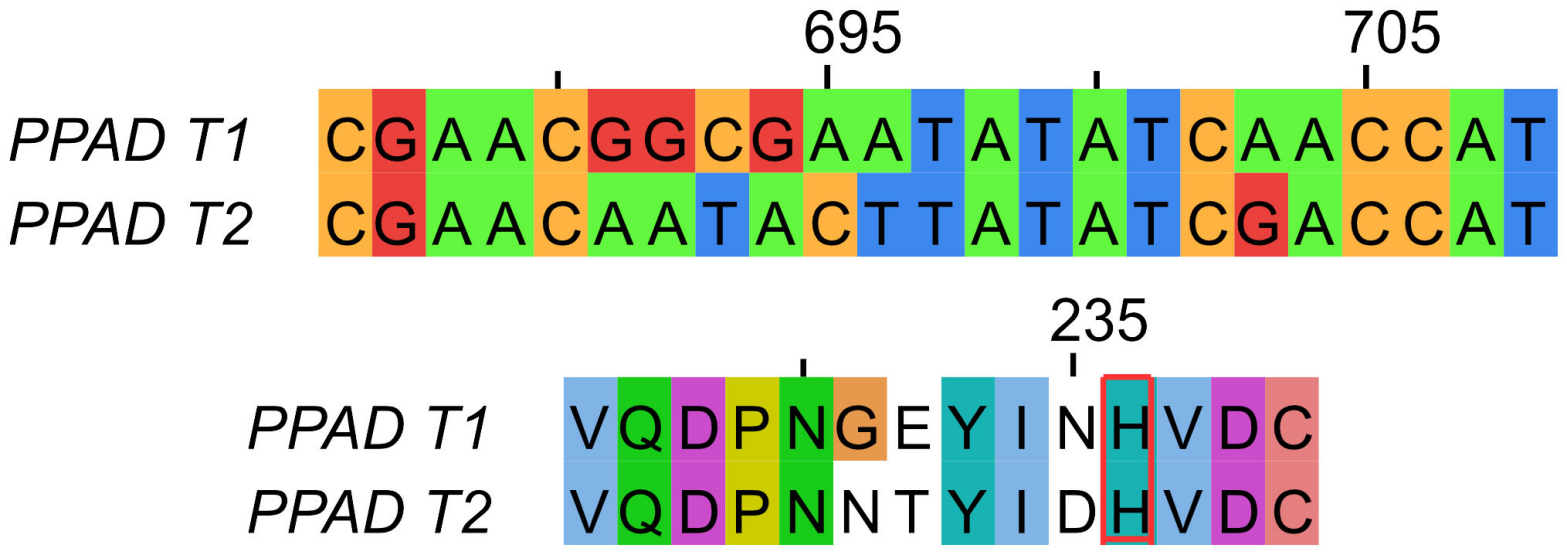
Comparison of PPAD gene (top) and protein (bottom) sequences from the reference laboratory strains ATCC 33277 and W83 (PPAD T1) and the triple polymorphic variant (G231N, E232T, N235D) (PPAD T2). The catalytic histidine residue is framed in red.

As previously demonstrated, 51 nucleotide changes were identified in the *ppad* gene sequence from the CP group, including 35 synonymous variants (68.63%) and 16 changes inducing modification of the sequence of the encoded protein. Among the latter group, 10 missense mutations (19.61%) and 6 polymorphic variants (11.76%) were found. Interestingly, 4 polymorphic variants (7.84%), but none of the missense mutations were found in close proximity to the active center of the PPAD. Further analyses revealed the presence of a new polymorphic variant inducing 3 amino acids substitution (G231N, E232T, N235D) near the active center of the PPAD protein in *P. gingivalis* strains cultured from 2 of the 4 subjects with moderate/advanced CP and all 5 subjects with advanced CP, accounting for ~30% of the subjects with CP (**Figure 3**). Interestingly, this polymorphic variant was absent from all other CP donors, classified with mild or moderate periodontitis.

### PPD and CAL values measured in gingival pockets within active inflammatory areas of periodontium correlate with the presence of *P. gingivalis* strain harboring a new polymorphic variant (G231N, E232T, N235D) of *ppad*

**Figure 4:**
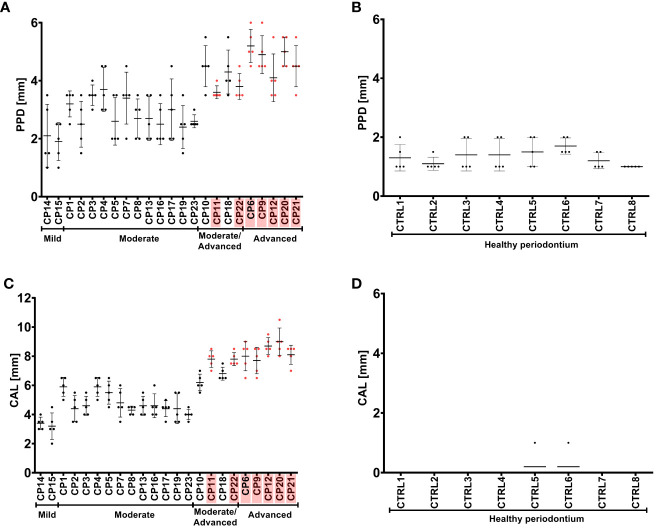
Pocket probing depth (PPD) **(A, B)** and clinical attachment loss (CAL) **(C, D)** of the periodontium of CP subjects **(A, C)** and healthy controls **(B, D)**. CP patients infected with *P. gingivalis* strains with a triple mutation in *ppad* (G231N, E232T, N235D) are marked in pink. Patients are grouped based on the severity of CP, as indicated below the graphs. Graphs show the mean with SD and raw measured values for each CP patient or control.

As previously described, *P. gingivalis* strains obtained from CP group were cultured from pooled GCF samples from five gingival pockets affected with active inflammation obtained from each subject. The PPD and CAL values measured in gingival pockets affected by inflammatory processes were significantly higher and correlated with the presence of *P. gingivalis* strains producing the new triple polymorphic variant (G231N, E232T, N235D) of *ppad* in CP donors diagnosed with advanced CP, and to some extent in moderate/advanced CP (**Figure 4**).

### PPAD activity of *P. gingivalis* clinical strains indicates a weak correlation with a G231N, E232T, N235D polymorphic variant

**Figure 5:**
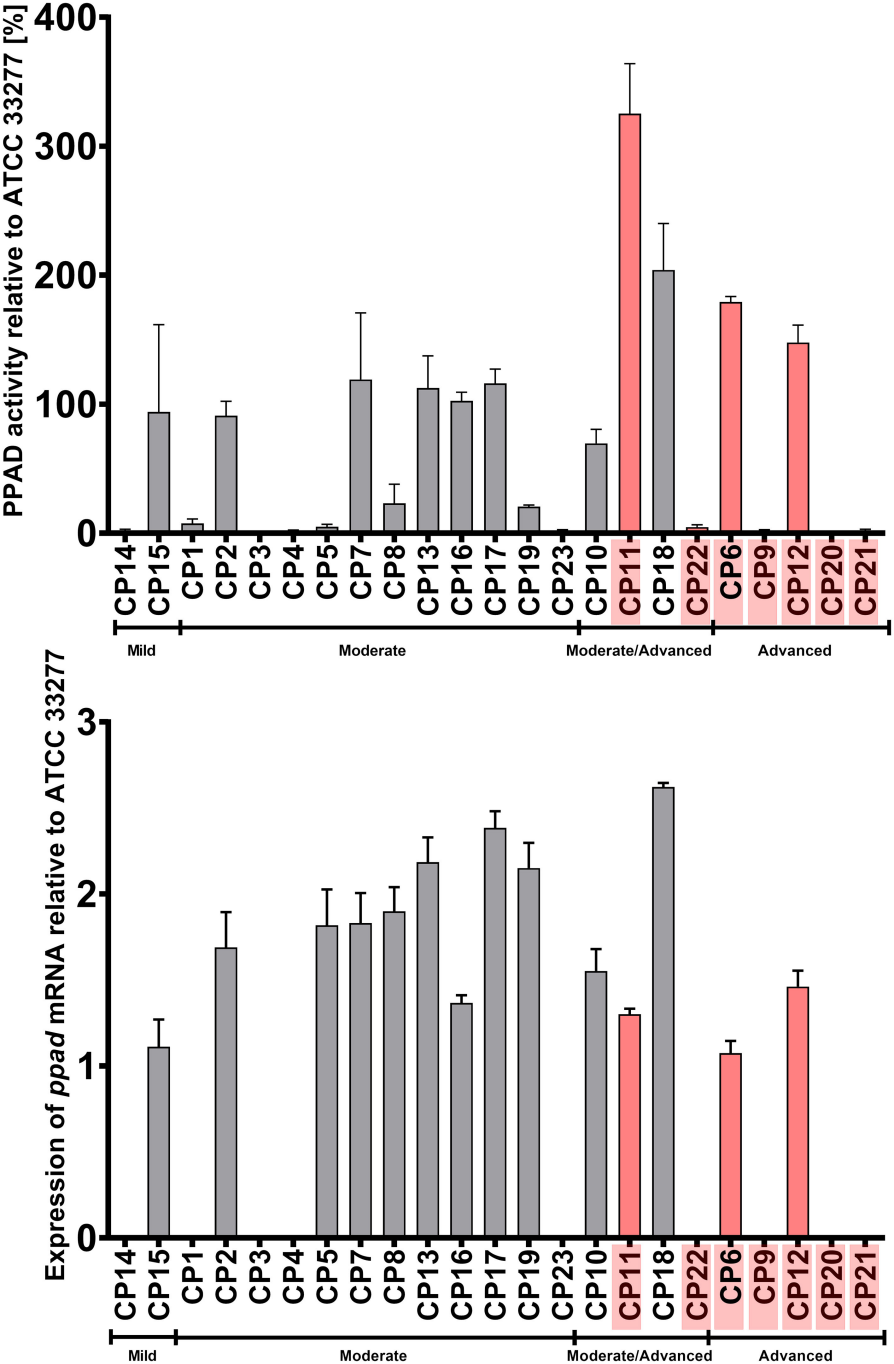
Activity (upper panel) and expression (lower panel) of PPAD in cultures of *P. gingivalis* strains isolated from CP patients. Samples were taken from liquid cultures of each strain, and enzyme activity and mRNA expression were calculated relative to the reference strain ATCC 33277. Data are shown as mean with SEM; CP patients infected with *P. gingivalis* strains harboring triple mutation in *ppad* (G231N, E232T, N235D) are marked in pink.

Evaluation of the activity of PPAD in the clinical strains yielded unclear results, as many *P. gingivalis* strains obtained from patients with mild and/or moderate CP displayed weak or nearly no PPAD activity. Compared with the ATCC 33277 reference strain, higher PPAD activity was observed in the strains obtained from two of the five patients with advanced periodontitis and two of the four with moderate/advanced periodontitis, including three strains that harbored the three amino acid substitutions (G231N, E232T, N235D) in close proximity to the active center of PPAD. The elevated PPAD activity in some strains could not be explained by an increase in *ppad* mRNA expression, as high mRNA expression in only one strain (CP18) corresponded to increased enzymatic activity. Interestingly, the expression level of the *ppad* gene with the triple mutation was generally lower than that of genes carrying a single amino acid substitution, an alteration abundant in moderate CP group (**Figure 5**).

### A laboratory construct of the triple variant, but no construct bearing a single amino acid substitution showed higher PPAD activity than the *P. gingivalis* ATCC 33277 type strain

**Figure 6:**
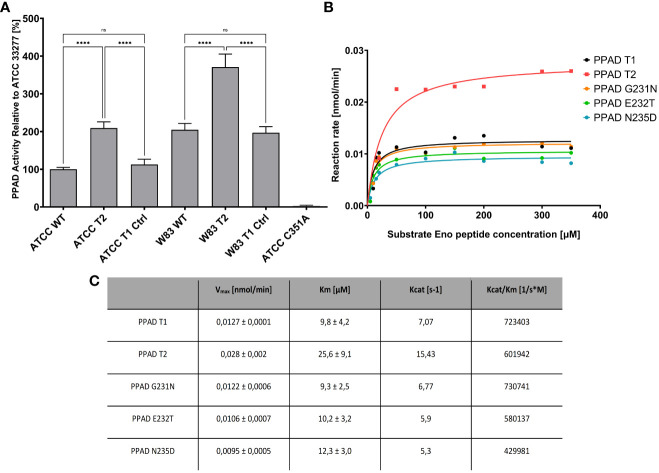
PPAD activity in *P. gingivalis* strains expressing mutated variants of PPAD and the purified enzymes with amino acid substitutions. **(A)** Strains of *P. gingivalis* ATCC 33277 and W83 were modified to code triple mutated PPAD T2 (with mutations G231N, E232T, N235D, strains ATCC T2 and W83 T2), control strains only with antibiotic resistance were also prepared (ATCC T1 Ctrl, W83 T1 Ctrl) and used for PPAD activity assay. A control ATCC strain with PPAD inactivated by mutation of catalytic cysteine residue C351A, was used as negative control. Results are presented as mean with SEM, **** p < 0.0001; ns, no statistical significance. **(B, C)** Enzyme kinetics of purified PPAD enzyme with no, single and triple amino acid substitutions. Enzymes were tested with fluorescently-labelled peptide and analyzed by HPLC; the table shows the kinetic parameters from Michaelis-Menten curves.

The properties of clinical isolates of *P. gingivalis* can differ due to genetic variations in this species, which can affect the production and/or secretion of virulence factors. To verify that the triple point mutations (G231N, E232T, N235D) in PPAD (called PPAD T2, and the reference sequence of PPAD from laboratory strains is called T1) are directly responsible for the increase in enzymatic activity, these mutations were introduced into the genomes of the reference strains ATCC 33277 and W83. Introduction of these mutations significantly increased their PPAD activity compared with their parental strains, even in W83 which already had higher activity than ATCC 33277 (**Figure 6A**). Purified PPAD enzyme with all three mutations showed higher enzymatic activity than the non-mutated enzyme, whereas single amino acid substitutions did not affect the activity of PPAD (**Figures 6B, C**).

## Discussion

The importance of *P. gingivalis* in the development and progression of CP is well established, with many studies showing that action of virulence factors produced by this species lead to the disease (5–7). By contrast, the concept of *P. gingivalis* involvement in rheumatoid arthritis (RA) remains still unclear and requires definitive, mechanistic proof (20, 23, 24, 40, 41). The bacterial factor that could be used to causatively link CP and RA is PPAD, the enzyme converting arginine into citrulline. The production of autoantibodies that react with citrullinated epitopes is a key process in RA, and PPAD can modify some of the proteins targeted by autoantibodies (15, 21, 24). This action may explain the association between bacteria-driven CP and the development of RA and further highlights the importance of PPAD as a pathological factor in both diseases. The action of PPAD as a virulence factor has been relatively well studied in the periodontal setting. This enzyme may have direct effects on host antibacterial defenses, exerted by the inactivation of complement component C5a impeding neutrophil antibacterial activity and contributing to biofilm formation and epithelial cell invasion (17, 42–45). PPAD can also modulate other inflammatory mechanisms; for example, an *in vitro* study has showed PPAD as the key factor stimulating *P. gingivalis*–infected gingival fibroblasts to produce PGE_2_ (18). The production of high levels of this inflammatory mediator can in turn trigger bone resorption by osteoclasts, resulting in the progressive destruction of periodontal tissues (46). Studies of PPAD in recent years have resulted in solving the crystal structure of this enzyme and explanation of its catalytic mechanism (14, 15, 38). Investigations of other aspects of PPAD biology revealed that it is a necessary virulence factor present ubiquitously in strains of *P. gingivalis* and is attached to the bacterial surface through the action of the type 9 secretion system, and that it can also be present on the surface of outer membrane vesicles (19, 34, 35, 47).

This study aimed to search for the correlation between a highly active, naturally occurring genetic variant of PPAD with three amino acid substitutions (G231N, E232T, N235D) and the virulence of *P. gingivalis (* 38). To this purpose, pooled samples of GCF from patients with CP were collected and cultivated to obtain clinical isolates of *P. gingivalis*. Because individual CP donors are likely colonized by a single strain of *P. gingivalis*, one strain per CP patient was obtained and its *ppad* gene sequence was analyzed (26).

This study identified a novel genetic variant (G231N, E232T, N235D) of PPAD, which was present in about 30% of the *P. gingivalis* strains analyzed and available in GenBank. The sequences in GenBank origin from different groups of CP patients from different countries, indicating that the presence of newly identified polymorphic variant of PPAD is ubiquitous and not restricted to any one population. Nevertheless, current data are insufficient to clearly show the correlation of the *ppad* variant with periodontitis mostly because CP-derived strains deposited in GenBank have incomplete data available.

Previous attempts to analyze *ppad* sequences in clinical isolates of *P. gingivalis* only identified mutations that were responsible for the localization of the enzyme or affected the process of secretion by the type 9 secretion system (35). A work recently published by Gabarrini et al. analyzing the entire *ppad* sequence to identify mutations, used very limited methodology, *i.e.*, restriction enzyme digestion analysis, and failed to detect noteworthy variances (34). On the contrary, the present study is based on more precise sequencing methodology to analyze variances of the single gene. Moreover, we evaluated here the clinical parameters of periodontitis in patients from whom the analyzed *P. gingivalis* strains were obtained. We showed that all CP patients infected with *P. gingivalis* strain harboring a novel genetic variant (G231N, E232T, N235D) of PPAD had more advanced clinical attachment loss than patients infected with the reference strain. Similarly, the values of pocket probing depth were higher in those patients, however this difference was not significant. These observations indicate that the presence of this genetic variant of PPAD in periodontal microflora may be associated with the progression of CP. The current data, however, are insufficient to determine whether strains bearing a novel PPAD variant induce disease progression or whether disease progression promotes colonization by these strains. Nevertheless, these results suggest that the PPAD type in *P. gingivalis* is associated with the severity of periodontitis, and may be recommended as a novel diagnostic tool of CP. Determination of the mechanism of PPAD activity connection with periodontitis progression requires certainly further investigation that would deliver important knowledge on periodontal tissue injury in the course of CP.

The present study also tested whether PPAD activity in naturally occurring strains of *P. gingivalis* with the novel variant of *ppad* differs from that in the ATCC reference strain. Although the activity of laboratory construct of novel *ppad* variant (T2) increased twofold in comparison to *P. gingivalis* ATCC 33277, colorimetric assays showed that PPAD activity in clinical strains cultured *in vitro* varied markedly (38). Most of these cultured strains showed very low levels of PPAD activity, perhaps due to the artificial conditions of *in vitro* culture, which may not be suitable for all *P. gingivalis* strains.

In contrast to PPAD activity, the expression levels of *ppad* mRNA were higher in most clinical isolates, and particularly from patients with moderate CP as compared to the reference ATCC 33277 strain, and the expression levels did not correlate with enzymatic activity. These observations may result from the effects of other genetic differences between analyzed clinical strains of *P. gingivalis* that were not identified in this study. Importantly, the W83 and ATCC 33277 strains used to express the engineered variant of *ppad* with three amino acid substitutions (T2) showed increased PPAD activity than the unmodified reference strain, but comparable levels of *ppad* mRNA arguing the mutations do not impact on gene expression. Because the clinical strains analyzed in the present study originated from patients diagnosed with CP, each strain was likely already adapted to specific conditions found in the diseased periodontium of individual patient.

The biochemical properties, including significantly increased enzymatic activity of a novel genetic variant (G231N, E232T, N235D) of PPAD corresponding to that identified in CP donors have been previously described (35, 38). Although previous report demonstrated the properties of this variant employing the reference ATCC 33277 strain, the present study used in this purpose the other reference *P. gingivalis* strain W83. PPAD activity was higher in wild-type W83 than in wild-type ATCC 33277, likely because of differences in their genetic background. The introduction of the PPAD variant bearing the three amino acid substitutions into W83 resulted in increased enzymatic activity compared with wild-type W83, similar to results observed with ATCC 33277 (38). Importantly in both cases, the insertion of an empty antibiotic cassette did not affect PPAD activity. Cumulatively these results suggest, that differences in the level of PPAD activity in different *P. gingivalis* strains depend on the enzymatic activity of the PPAD variants.

A previous study suggested that each point mutation of a novel triple variant may have different effects that cumulatively increase the activity of PPAD (38). Therefore, further we investigated the effects of each of the point mutations introduced separately into the *ppad* gene. Kinetic analysis using purified PPAD proteins showed that introduction of each of the individual mutations (G231N, E232T or N235D) had no effect on enzyme activity. This observation suggests that introduction of all three mutations was required to have a measurable effect on enzyme activity, although each may alter protein stability or expression.

However, our finding has several limitations due to small sample size, the gender bias (the CP group has more women, and the control group has more men), age disparities between the CP and the healthy control groups group, the lack of detailed information on the general health criteria for the control group and the reliance on enzymatic activity as the sole indicator of *P. gingivalis* virulence. Also, the study participants were enrolled and the samples were collected before 2017, and hence in the study, CP classification from 1999 was used, including PPD and CAL parameters calculation. Virulence is a multifactorial trait influenced by various factors, and focusing solely on enzymatic activity may not capture the full complexity of *P. gingivalis* pathogenicity. These confines raise concerns about the study’s statistical power, internal validity, and the generalizability of findings. Nevertheless, the presented results provide initial insights into the plausible relationship between the *ppad* gene variability and clinical characteristics of chronic periodontitis.

## Conclusions

Sequencing of the *ppad* gene showed that *P. gingivalis* strains isolated from individual patients with CP varied markedly. About 30% of these CP patients, who were diagnosed with advanced or moderate/advanced periodontitis, harbored *P. gingivalis* strains with a new polymorphic variant (G231N, E232T, N235D) in the *ppad* gene. Biochemical tests of a laboratory construct of this variant showed that its enzymatic activity was greater as compared to the wild-type *P. gingivalis* strain. These findings, together with the clinical characteristics of the periodontium of CP patients infected by strains bearing this variant, suggest an indirect effect of a new PPAD mutant on bacterial virulence. These observations, although preliminary and with limitations, suggest a need for further investigations of PPAD as a risk factor for developing severe CP and its association with RA.

## Data availability statement

The original contributions presented in the study are included in the article/supplementary materials, further inquiries can be directed to the corresponding author.

## Ethics statement

The studies involving humans were approved by Bioethics Committee of Jagiellonian University Medical College in Krakow, Poland. The studies were conducted in accordance with the local legislation and institutional requirements. The participants provided their written informed consent to participate in this study.

## Author contributions

GB: Data curation, Formal analysis, Investigation, Methodology, Software, Writing – original draft. KS: Formal analysis, Methodology, Software, Validation, Writing – original draft. KŁ-B: Data curation, Formal analysis, Investigation, Methodology, Writing – review & editing. AD-K: Data curation, Formal analysis, Software, Visualization, Writing – original draft. ZN: Data curation, Formal analysis, Investigation, Methodology, Writing – original draft. AK: Data curation, Formal analysis, Software, Visualization, Writing – review & editing. EB: Formal analysis, Investigation, Methodology, Validation, Writing – review & editing. TKan: Formal analysis, Validation, Visualization, Writing – review & editing. AG: Data curation, Formal analysis, Writing – review & editing. TKac: Methodology, Visualization, Writing – review & editing. MC-G: Conceptualization, Data curation, Supervision, Writing – review & editing. JP: Conceptualization, Data curation, Resources, Supervision, Validation, Writing – review & editing. KG: Conceptualization, Data curation, Investigation, Methodology, Project administration, Resources, Supervision, Validation, Writing – review & editing.
