# Supplementary material for: Identification of a new genetic variant (G231N, E232T, N235D) of peptidylarginine deiminase from P. gingivalis in advanced periodontitis
Source: Front Immunol. 2024 Mar 21;15:1355357. doi: 10.3389/fimmu.2024.1355357 (PMC10991804; doi:10.3389/fimmu.2024.1355357)
Supplement: Supplementary file 1 [file Table_1.docx]

| GenBank accession numbers of new *ppad* sequences obtained in this study and deposited in the database | | Accession numbers of GenBank sequences analyzed in this study |
| --- | --- | --- |
| Strain name | Accession number |  |
| CP1 | PP079918 | FUFB01000005 |
| CP2 | PP079919 | FUFC01000005 |
| CP3 | PP079920 | FUFD01000013 |
| CP4 | PP079921 | FUFE01000062 |
| CP5 | PP079922 | FUGG01000063 |
| CP6 | PP079923 | FUFG01000025 |
| CP7 | PP079924 | CP012889 |
| CP8 | PP079925 | KP862656 |
| CP9 | PP079926 | KP862650 |
| CP10 | PP079927 | CP013131 |
| CP11 | PP079928 | FUFF01000006 |
| CP12 | PP079929 | CP011995 |
| CP13 | PP079930 | FUFJ01000053 |
| CP14 | PP079931 | CP011996 |
| CP15 | PP079932 | BCBV01000036 |
| CP16 | PP079933 | AP009380 |
| CP17 | PP079934 | FUFH01000058 |
| CP18 | PP079935 | AWVC01000009 |
| CP19 | PP079936 | AWVD01000025 |
| CP20 | PP079937 | AWUU01000015 |
| CP21 | PP079938 | AWUV01000067 |
| CP22 | PP079939 | AWUW01000017 |
| CP23 | PP079940 | NZ_CP007756 |
| CTRL1 | PP079941 | APMB01000037 |
| CTRL2 | PP079942 | KP862652 |
| CTRL3 | PP079943 | KP862653 |
| CTRL4 | PP079944 | KP862654 |
| CTRL5 | PP079945 | KP862655 |
| CTRL6 | PP079946 | KP862651 |
| CTRL7 | PP079947 | LOEL01000042 |
| CTRL8 | PP079948 | ASYL01000006 |
|  |  | AP012203 |
|  |  | AJZS01000007 |
|  |  | AWVE01000083 |
|  |  | FUFI01000021 |
|  |  | NC_002950 |

**Table 1** List of sequences obtained in this study and deposited in the GenBank database and sequences from the database used in this study.
